# Vitamin D Supplementation Influences Ultramarathon-Induced Changes in Serum Amino Acid Levels, Tryptophan/Branched-Chain Amino Acid Ratio, and Arginine/Asymmetric Dimethylarginine Ratio

**DOI:** 10.3390/nu15163536

**Published:** 2023-08-11

**Authors:** Jan Mieszkowski, Paulina Brzezińska, Błażej Stankiewicz, Andrzej Kochanowicz, Katarzyna Zolodkiewicz, Bartłomiej Niespodziński, Joanna Reczkowicz, Tomasz Kowalik, Tomasz Waldziński, Jędrzej Antosiewicz

**Affiliations:** 1 Department of Gymnastics and Dance, Gdańsk University of Physical Education and Sport, 80-336 Gdańsk, Poland; ppb.brzezinska@gmail.com (P.B.); andrzejkochanowicz@o2.pl (A.K.); zoladkiewicz.k@gmail.com (K.Z.); 2 Faculty of Physical Education and Sport, Charles University, 162 52 Prague, Czech Republic; 3 Department of Theory and Methodology of Physical Education and Sport, Faculty of Health Sciences and Physical Education, Kazimierz Wielki University, 85-064 Bydgoszcz, Poland; balzej1975@interia.pl (B.S.); tomkow79@gmail.com (T.K.); 4 Department of Biomedical Basis of Physical Education, Faculty of Health Sciences and Physical Education, Kazimierz Wielki University, 85-064 Bydgoszcz, Poland; bartlomiej.niespodzinski@ukw.edu.pl; 5 Department of Bioenergetics and Physiology of Exercise, Medical University of Gdańsk, 80-211 Gdańsk, Poland; joanna.reczkowicz@gumed.edu.pl; 6 Faculty of Health Sciences, University of Lomza, 14 Akademicka Street, 18-400 Lomza, Poland; twaldzinski@ansl.edu.pl

**Keywords:** ultramarathon, skeletal muscle damage, amino acid, ADMA, SDMA

## Abstract

Exercise affects serum levels of amino acids and their metabolites, with important metabolic consequences. Since vitamin D impacts skeletal muscle protein degradation, we hypothesised that it would also impact exercise-induced changes in serum amino acid levels and the serum levels of arginine metabolites, influencing the body’s ability to synthesise NO. Accordingly, we analysed the effect of a single high-dose vitamin D supplementation on the serum levels of various amino acids in ultramarathon runners. Thirty-five male amateur runners were assigned to the supplemented group, administered 150,000 IU vitamin D in vegetable oil 24 h before the run (*n* = 16), or the control (placebo) group (*n* = 19). Blood was sampled 24 h before, immediately after, and 24 h after the run. Changes in the serum levels of some amino acids were distinct in the two groups. The asymmetric dimethyl arginine levels were significantly decreased immediately after the run and increased 24 h later and were not affected by the supplementation. The symmetric dimethyl arginine levels were increased after the run in both groups but were lower in the supplemented group than in the placebo group 24 h after the run. The dimethylamine levels increased significantly in the supplemented group as compared to the placebo group. In conclusion, vitamin D impacts exercise-induced changes in serum amino acids and methylated arginine metabolites.

## 1. Introduction

According to an increasing number of studies, vitamin D sufficiency positively influences skeletal muscle strength and endurance performance [1,2,3]. For example, vitamin D supplementation augments leg lean mass increase after Nordic walking training in women [1]. Conversely, low vitamin D_3_ active metabolite serum levels are associated with lower isometric arm strength [2]. Furthermore, in humans, adaptive changes in the heart, such as increased left ventricular volume, are blunted in subjects with low 25(OH)D_3_ levels, confirming the role of vitamin D in adaptation to endurance training [3]. However, despite increasing knowledge of the importance of vitamin D among athletes and physically active individuals, many of them are vitamin-D-deficient, especially in Northern countries [4,5,6,7].

The active metabolite of vitamin D, 1,25(OH)_2_D_3_ [8,9,10], regulates actions on 1000 genes by binding to its specific nuclear receptor, the vitamin D receptor (VDR). VDRs are expressed in muscle tissue at various stages of embryonic development, when myoblasts fuse to form myotubes [11]. The non-genomic effect of vitamin D is much faster and results from its combination with VDRs located on the cell membrane. As a result of binding with the membrane receptor, 1,25(OH)_2_D_3_ activates several alternative messenger pathways (conversely, low vitamin D_3_ active metabolite serum levels are associated with lower isometric arm strength, ERK-1/2 cascades, phospholipase C, and c-myc), which cause cellular effects within a few to several seconds from activation. Additionally, the binding of vitamin D to its specific nuclear receptor stimulates the intracellular uptake of inorganic phosphates (necessary for the muscle contractility), which may influence myostatin inhibition and induces the expression of myoblast determination protein 1 (MyoD1) [12]. Furthermore, it affects many other muscle intercellular pathways, such as forkhead box O (FOXO) 3, Notch signalling, myoblast self-renewal, and many others [12]. It is worth noting that vitamin D activity is mainly regulated by its serum concentration; most of the data suggest that 25(OH)D_3_ levels above 30 ng/mL/35 ng/mL provide optimal musculoskeletal benefits, and levels under <25 nmol/L are associated with significantly lower musculoskeletal effectiveness [13,14,15] and muscle strength [1].

Nevertheless, the effects of vitamin D on muscle and human performance still remain to be completely understood. In addition to the proven effect on muscle tissue, there are still many pathways that may be affected by vitamin D and its activity.

It is well-established that endurance exercise capacity is determined by working muscle blood flow, which is determined by cardiac output and microvascular blood flow [16,17]. The latter is tightly regulated and depends on the synthesis of nitric oxide (NO), which is involved in the relaxation of the vasculature of smooth muscle cells [4]. NO is synthesised from arginine by endothelial NO synthases (eNOS) in a reaction that also produces citrulline. Arginine availability is an important determinant of NO synthesis. In many cells, including arterial endothelial cells, two enzymatic reactions recycle citrulline into arginine. The importance of these reactions is underlined by the observation that in vitro, citrulline stimulates NO production even in a medium containing saturating arginine levels [17]. Furthermore, arginine availability can be determined by measuring the activity of arginase, the enzyme that converts arginine into urea and ornithine. In addition, there is overwhelming evidence showing that arginine supplementation does not improve eNOS activity, and no ergogenic effects are observed [17]. Hence, NO production depends mainly on the efficient recycling of arginine-derived citrulline back into arginine and not so much on an exogenous arginine supply. NO synthesis is also controlled by asymmetric dimethylarginine (ADMA), an endogenous inhibitor of NOS, and possibly by symmetric dimethylarginine (SDMA), which alters arginine concentration through competition for amino acid transporters [18]. A considerable amount of ADMA is further metabolised into citrulline and dimethylamine (DMA) in a reaction catalysed by dimethylarginine dimethylaminohydrolase (DDAH), with approximately 20% of the ADMA excreted by the kidneys [19]. In conditions such as oxidative stress, hyperhomocysteinemia, hypertension, and others, DDAH activity has been reported to decrease [19]. 

Vitamin D has been reported to exert some antioxidative effects [20]. For example, vitamin D supplementation reduces oxidative damage to proteins and lipids in the skeletal muscle of patients with low back pain [20]. However, many properties of vitamin D still need to be fully defined, and the complexity of its impact is a research problem requiring many studies and analyses. Thus, the current study aimed to evaluate the impacts of high-dose vitamin D supplementation on ultramarathon-induced changes in the levels of amino acids and their metabolites. We hypothesised that improved vitamin D status would impact the serum levels of arginine metabolites, influencing the body’s ability to synthesise NO. 

## 2. Materials and Methods

### 2.1. Experimental Overview 

This study is a continuation of a previous investigation [21] that aimed to assess the impact of a single high dose of vitamin D on vitamin D metabolites in ultramarathon runners and is a part of the project “Vitamin D as a Factor Modifying Adaptation to Exercise”. The study was registered as the clinical trial NCT03417700.

The study was designed as a double-blind randomised controlled trial with parallel groups, i.e., the supplementation and placebo (control) groups. The supplementation involved the administration of a single high dose of vitamin D. 

### 2.2. Participants 

A group of thirty-five semi-professional male ultramarathon runners took part in the study. All participants started in the Lower Silesian Mountain Runs Festival 2018 Ultra Marathon Race (characteristic—Supplementary Materials). They were randomly assigned to two groups: the experimental (supplemented, S; *n* = 16) or placebo (control, C; *n* = 19) group. All the runners had previous ultramarathon experience (a minimum of 5 starts). Detailed information about the participants’ physical characteristics, training loads, and performance in the Cooper test [22] is provided elsewhere [1].

The study protocol was approved by the Bioethics Committee for Clinical Research at the Regional Medical Chamber in Gdańsk (decision no. KB-24/16). The study was registered as the clinical trial NCT03417700. 

### 2.3. Ultramarathon Run

All participants participated in the Lower Silesian Mountain Run Festival 2018 organised at Lądek Zdrój (Lower Silesian Voivodeship, Poland), with a maximum course length of 240 km. 

### 2.4. Vitamin D Supplementation

The supplemented group were given a single high dose (150,000 IU) of vitamin D_3_ as a solution in 10 mL of vegetable oil 24 h before starting the ultramarathon. The placebo group received an equivalent volume of placebo solution, administered in the same way as the vitamin D in the S group. 

### 2.5. Sample Collection

Blood samples for serum analysis were taken by a medical diagnostic professional according to the experimental protocol at three different time points: 24 h before the run, immediately after the run (within 5 min of finishing the run), and 24 h after the run. The blood was collected in Sarstedt S-Monovette tubes (S-Monovette^®^ Sarstedt AG&Co, Nümbrecht, Germany) containing a coagulation accelerator. The serum was portioned and frozen at −80 °C until analysis.

For the amino acid analysis, we used a modified method developed by Waraksa et al. 2019 [9]. Serum proteins were first precipitated and derivatized. Quantitative analysis was performed using liquid chromatography–tandem mass spectrometry (Shimadzu Nexera X2 UHPLC (Shimadzu, Japan)) coupled with an 8050 triple-quadruple detector (Shimadzu, Japan). The raw data were collected, processed, and quantified using LabSolutions LCGC.

### 2.6. Statistical Analysis

The minimal total sample size for a medium effect size at the power of 0.8 and significance level of 0.05 was calculated using GPower ver. 3.1.9.2. and provided a minimal population of 28 subjects.

All data are presented as the mean ± standard deviation. To assess the impact of the ultramarathon combined with vitamin D supplementation on amino acid levels, a two-way (2 × 3) analysis of variance (ANOVA) with repeated measures was performed. The group (S, supplemented; C, control) was the first factor and was represented by participants administered a high dose of vitamin D or placebo, accordingly. The second factor was the time point concerning the ultramarathon run completion: I, 24 h before the run; II, immediately after the run; and III, 24 h after the run. If a significant interaction effect of the two factors was observed, Tukey’s post hoc test was performed.

The assumptions of ANOVA, a normal distribution (Shapiro–Wilk test), and homogeneity of variance (Levene test) were verified and held. 

To evaluate the magnitude of the effect, the eta-squared statistic (η^2^) was used, wherein values equal to or less than 0.01, 0.06, and 0.14 and those over 0.14 indicated a trivial, small, moderate, and large effect, respectively. The level of significance for all tests was set at α = 0.05. The required sample size of 28 participants for a medium effect size and power of 0.80 was determined using G*Power software ver. 3.1.9.4 (Franz Faul et al., Universität Kiel, Kiel, Germany). 

## 3. Results

**Figure 1:**
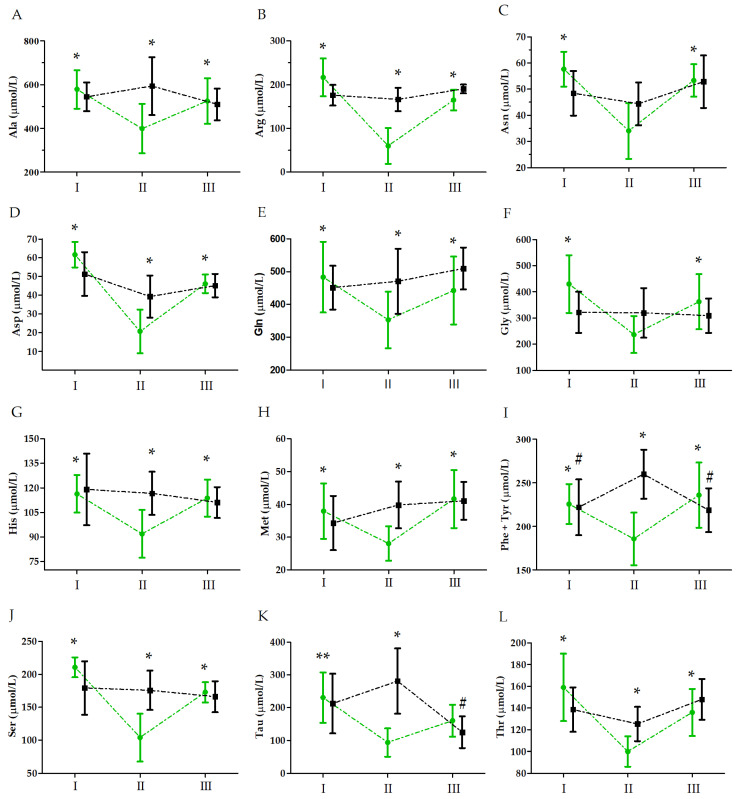
Ultramarathon-induced changes in amino acid levels in runners who received a single high dose of vitamin D (group S, green) and runners who received the placebo (group C, black). (**A**) Alanine (Ala); (**B**) arginine (Arg); (**C**) asparagine (Asn); (**D**) aspartic acid (AI); (**E**) glutamic acid (Gln); (**F**) glutamine (Gly); (**G**) histidine (His); (**H**) methionine (Met); (**I**) phenylalanine + tyrosine (Phe + Tyr); (**J**) serine (Ser); (**K**) taurine (Tau); (**L**) threonine (Thr). Sampling: I, 24 h before the run; II, immediately after the run; and III, 24 h after the run. Abbreviations: *, significant difference vs. group S immediately after the run; **, significant difference vs. group S immediately after and 24 h after the run; #, significant difference vs. group C immediately after the run. The significance level was set at *p* < 0.01.

The analysis and changes in amino acid levels induced by the ultramarathon run are presented in Table 1 and Figure 1, respectively. The two-factor ANOVA with repeated measures revealed a significant effect of the UM factor on all the amino acids, except for Ala and Phe + Tyr. In turn, significant interactions of the GR × UM factor were noted for Ala, Arg, Asn, Asp, Gln, Gly, His, Met, Phe + Tyr, Ser, and Thr. Post hoc analysis revealed a significant decrease of these amino acid levels immediately after the run relative to the baseline values in the supplemented group. No significant changes were observed in the control group, except for Tau and Phe + Tyr, where a significant increase immediately after the run was noted. In both groups, the levels of the tested amino acids were close to the baseline values 24 h after the run.

**Figure 2:**
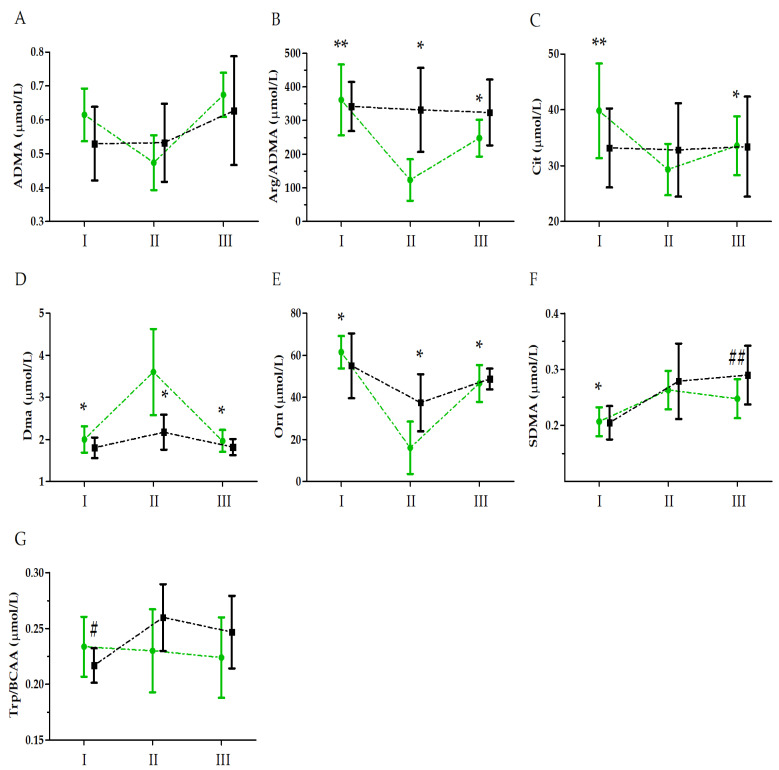
Ultramarathon-induced changes in amino acid metabolite levels in runners who received a single high dose of vitamin D (group S, green) and runners who received the placebo (group C, black). (**A**) Asymmetric dimethylarginine (ADMA); (**B**) arginine to asymmetric dimethylarginine ratio (Arg/ADMA); (**C**) citrulline (Cit); (**D**) dimethylamine (DMA); (**E**) ornithine (Orn); (**F**) symmetric dimethylarginine (SDMA); (**G**) tryptophan to branched-chain amino acid ratio (Trp/BCAA). Sampling: I, 24 h before the run; II, immediately after the run; and III, 24 h after the run. Abbreviations: *, significant difference vs. group S immediately after the run; **, significant difference vs. group S immediately after and 24 h after the run; #, significant difference vs. group C immediately after the run; ##, significant difference vs. group C 24 h after the run. The significance level was set at *p* < 0.01.

Analysis of the two-factor mixed ANOVA model also revealed an effect of UM and UM × GR interactions on the amino acid metabolites (Table 2). The post hoc analysis indicated a decrease in the Arg/ADMA, citrulline, and ornithine levels relative to the baseline values in the supplemented group, with no significant changes in the control group (Figure 2). 

The dimethylamine (DMA) and symmetric dimethylarginine (SDM) ratio increased relative to the baseline values in the supplemented group, with no changes in the control group. On the other hand, no major changes in the Trp/BCAA ratio were apparent in the supplemented group, with a notable increase immediately after the run compared to the baseline values in the control group.

## 4. Discussion

In the current study, we demonstrated for the first time that supplementation with vitamin D 24 h before an ultramarathon run significantly influences serum levels of arginine, DMA, citrulline, and ornithine. Notably, arginine levels did not change in athletes not supplemented with vitamin D. 

Serum arginine levels reflect arginine formation from citrulline and ornithine and its consumption during exercise. Previously, a decrease in serum arginine levels was observed after a triathlon; however, neither Cit nor Ort levels were measured in that study [23]. Conversely, in another study, while 120 min of exercise on a cycle ergometer did not affect serum arginine levels, a decrease was observed in athletes whose Arg levels were increased before the exercise by supplementation with an amino acid mixture [21]. Notably, vitamin D status was not evaluated in the cited studies. 

In the current study, we observed that vitamin D supplementation was associated with exercise-induced changes in serum amino acids and their metabolites. 

We were not able to determine the mechanism through which vitamin D affects arginine levels during such an exhausting run. However, the significantly increased serum Phe + Tyr levels after the run in the placebo-supplemented athletes indicate that exercise stimulates proteolysis [22]. Accordingly, several amino acids, including Arg, could be exported into the bloodstream from the skeletal muscle and possibly other tissues. In the vitamin-D-supplemented athletes, the Phe + Tyr levels 24 h post-race were lower than those in the placebo group. This indicates that vitamin D may block proteolysis, as suggested previously [24]. 

Protein degradation products include methylated forms of arginine, such as SDMA and ADMA. Under physiological conditions, some proteins that contain Arg are methylated by protein arginine methyltransferases. This can lead to the formation of proteins with two methyl groups at one nitrogen atom of the guanidino group (ADMA) or Arg with one methyl group on each terminal guanidine nitrogen atom (SDMA). Both free ADMA and SDMA are released following proteolysis. Approximately 300 μmol of ADMA is formed in healthy adults, with 20% excreted by the kidneys and the remainder of the metabolised excreted by DDAH [25]. It is not known whether extreme exercise affects the metabolic fate of ADMA. Here, we observed that while ADMA levels decreased immediately after the run in both groups, they increased 24 h later. A similar observation was made in triathletes [23]. Notably, we observed a significant difference in the serum DMA levels in the two groups, which were higher in the supplemented group. This indicated that the reaction catalysed by DDAH was augmented by vitamin D. Similar to SDMA, DMA elimination is mainly dependent on urine excretion [26]. Immediately after the run, the serum SDMA levels increased in both groups, indicating that the urine excretion of SDMA was unaffected by vitamin D supplementation. Hence, the increase in DMA levels could result from increased DMA formation rather than impaired excretion. Conversely, 24 h after the run, the DMA levels returned to baseline, while the SDMA levels in the supplemented athletes were lower than those in the placebo group, suggesting better urine elimination in the supplemented group. 

Vitamin D supplementation also significantly affected ultramarathon-induced changes in the serum levels of amino acids. The levels of such amino acids as serine, alanine, leucine, isoleucine, valine, methionine, and others decreased after the run only in the athletes in the supplemented group. These amino acids were metabolised during the run. Hence, the observed decrease in their levels in the vitamin-D-supplemented athletes and lack of change or increase in the placebo group could be explained by the vitamin-D-induced inhibition of proteolysis. A significant decrease in essential and non-essential amino acid levels after a half-ironman triathlon was reported previously [27]. Meanwhile, similar to our observations of the vitamin-D-supplemented group in the current study, Areces et al. [27] did not evaluate the vitamin D status of their study participants. However, the study was performed in Spain, and thus, one could expect that the athletes’ serum vitamin D levels were sufficient. 

Vitamin D modulates changes in kynurenine metabolism induced by endurance exercise [28]. However, it is essential to note that Trp can also be metabolised to 5-hydroxy-tryptamine (serotonin). The formation of serotonin in the brain might play a role in the experience of fatigue during and after physical exercise. Tryptophan hydroxylase limits the synthesis of serotonin. Because this enzyme is not saturated with a substrate, the rate of serotonin synthesis is sensitive to changes in blood tryptophan levels and its transport across the blood–brain barrier. Trp is transported to the brain via a transporter that also handles other large neutral amino acids, including branched-chain amino acids (BCAAs). Accordingly, rats given low doses of Trp perform better than those given high doses of Trp [29]. Conversely, sustained exercise increases the plasma ratio of free tryptophan to BCAA in animals, the uptake of tryptophan by the brain (in humans), and the synthesis and release of serotonin in the rat brain [30]. Thus, it has been postulated that a decrease in serum BCAA levels during exercise and an increase in the serum Trp/BCAA ratio observed after endurance exercise can augment Trp transport to the brain [31]. Consequently, serotonin formation in the central nervous system increases, resulting in fatigue. Notably, in the current study, the Trp/BCAA ratio did not change in the athletes in the supplemented group, while it increased in the control group. This indicates that vitamin D could be involved in reducing fatigue during exercise. Consistently, BCAA supplementation before a marathon increases serum amino acid levels and provides some improvements in running performance [31]. 

Collectively, the current study revealed distinct serum levels of some amino acids, including tyrosine and phenylalanine, after an ultramarathon race, which is noteworthy in light of the applied supplementation. The mechanisms underlying these effects may be multifaceted. The results suggest that an intake of vitamin D could suppress the protein degradation increase that occurs during sustained exercise. Furthermore, the Arg/ADMA ratio was lower in the vitamin-D-supplemented athletes than in the placebo group, despite the observed increase in DMA levels, indicating rapid ADMA metabolism. It is possible that the serum Arg/ADMA ratio could be different from that in tissues, as suggested above [19,32]. Conversely, the unchanged serum Trp/BCAA ratio in runners supplemented with vitamin D might have contributed to their improved physical performance. 

### 4.1. Limitations

While this study concentrated on the distinct serum levels of amino acids following an ultramarathon race and the potential impacts of vitamin D supplementation, it has certain limitations. Despite our meticulously designed procedures, we were unable to analyse the concentrations of methylarginines in the subjects’ urine. These urinary levels could have provided further insight into the efficiency of methylarginine elimination by the kidneys and offered an enhanced understanding of their metabolism and renal function. However, the data we gathered significantly elucidate the range of pathophysiological processes associated with these compounds, particularly in the context of the physiological stress induced by an ultramarathon. This study provides substantial value for understanding physiological responses to extreme endurance events with respect to these compounds and their role in extreme physical exertion.

### 4.2. Conclusions

Demonstrating the direct effects of vitamin D supplementation on ultramarathon-induced changes in serum amino acid levels can prove that effective vitamin D supplementation (which is associated with an increased blood concentration of vitamin D metabolites) could suppress protein degradation. Some evidence for the presented activity of vitamin D in muscle protein turnover was already known almost forty years ago, for example, in Wassner et al.’s work [33]. These authors proved that muscle protein turnover rates (both in vivo and in vitro) and myofibrillar protein degradation are determined by vitamin D status, which is increased in cases of vitamin D deficiency. Moreover, vitamin-D-supplemented rats showed significantly higher insulin levels (probably due to the role of vitamin D in maintaining plasma calcium), and it may affect muscle protein turnover by preventing hypocalcaemia and directly stimulating insulin secretion [33], suggesting that the observed effect of vitamin D on health can be multi-directional.

Over the years, many researchers have proven that vitamin D supplementation can inhibit muscle protein degradation caused by external factors, ageing, diseases, or, essentially, demanding exercises [34,35,36]. This is why supplementation with vitamin D, especially in cases where the vitamin D status is insufficient, can benefit the athlete’s health, especially in demanding and long-lasting exercises like ultramarathon mountain running. The main pathways of this process are still not fully understood, and the presented data, concentrating only on the distinct serum levels of amino acids following an ultramarathon race, provide only a few answers regarding the interdependence and interactions of vitamin D status and post-exercise metabolic changes. 

However, considering the multi-directional activity of vitamin D (from the inhibition of inflammation to metabolic regulation, body homeostasis, mitochondrial activation, and hormonal regulation), its appropriate status in sports competition seems to be very beneficial or even essential to maintaining good health. This study provides substantial value for understanding physiological responses to extreme endurance events with respect to these compounds and their role in extreme physical exertion.

## Figures and Tables

**Table 1:** Two-way ANOVA (2 groups × 3 repeated measures) of changes in Please verify that the intended meaning is retained. Amino acid levels induced by the ultramarathon.

Variable	Effect	F	Df	*p*	Effect Size (η^2^)	Post Hoc Outcome
Alanine (Ala)	GR	1.46	1, 33	0.24	0.10	
	UM	2.30	2, 66	0.12	0.16	
	GR × UM	8.34	2, 66	0.01 **	0.41	SI > SII, SIII; CII < SII
Arginine (Arg)	GR	4.07	1, 33	0.66	0.25	
	UM	79.84	2, 66	0.01 **	0.86	II < I, III; I > III
	GR × UM	57.15	2, 66	0.01 **	0.82	SI > SII; CII < SII
Asparagine (Asn)	GR	0.02	1, 33	0.86	0.01	
	UM	17.98	2, 66	0.01 **	0.59	II < I, III
	GR × UM	8.04	2, 66	0.01 **	0.40	SII < SI, SIII
Aspartic acid (Asp)	GR	0.40	1, 33	0.53	0.03	
	UM	50.57	2, 66	0.01 **	0.80	II < I, III; I > III
	GR × UM	15.50	2, 66	0.01 **	0.56	SII < SI, SIII; CII < SII
Cysteine (Cys)	GR	11.05	1, 33	0.01 **	0.47	S > C
	UM	3.84	2, 66	0.03 *	0.24	II < I
	GR × UM	1.10	2, 66	0.34	0.08	
Glutamic acid (Glu)	GR	2.49	1, 33	0.14	0.17	I > III
	UM	3.65	2, 66	0.04 *	0.23	
	GR × UM	0.68	2, 66	0.51	0.05	
Glutamine (Gln)	GR	1.29	1, 33	0.27	0.09	
	UM	7.53	2, 66	0.01 **	0.38	II < I, III
	GR × UM	9.05	2, 66	0.01 **	0.43	SII < SI, SIII
Glycine (Gly)	GR	0.21	1, 33	0.65	0.01	
	UM	14.04	2, 66	0.01 **	0.53	II < I, III
	GR × UM	14.03	2, 66	0.01 **	0.53	SII < SI, SIII
Histidine (His)	GR	1.54	1, 33	0.11	0.11	
	UM	9.80	2, 66	0.44	0.44	II < I, III
	GR × UM	11.34	2, 66	0.48	0.48	SII < SI, SIII; CII < SII
Isoleucine (Ile)	GR	0.12	1, 33	0.72	0.01	II < I, III
	UM	10.31	2, 66	0.01 **	0.46	
	GR × UM	1.73	2, 66	0.19	0.12	
Leucine (Leu)	GR	0.75	1, 33	0.78	0.01	II < I, III
	UM	12.99	2, 66	0.01 **	0.51	
	GR × UM	2.18	2, 66	0.13	0.15	
Lysine (Lys)	GR	0.89	1, 33	0.36	0.06	II < I, III
	UM	9.90	2, 66	0.01 **	0.45	
	GR × UM	1.70	2, 66	0.20	0.12	
Methionine (Met)	GR	0.89	1, 33	0.36	0.06	
	UM	4.22	2, 66	0.02 *	0.26	II < III
	GR × UM	2.86	2, 66	0.01 *	0.28	SII < SI, SIII; CII < SII
Phenylalanine Tyrosine (Phe + Tyr)	GR	2.85	1, 33	0.11	0.19	
	UM	0.54	2, 66	0.58	0.04	
	GR × UM	27.26	2, 66	0.01 **	0.69	SII < SI, SIII; CII > CI, CIII; CII > SII
Proline (Pro)	GR	1.17	1, 33	0.30	0.08	
	UM	13.21	2, 66	0.01 **	0.52	I > II, III
	GR × UM	0.74	2, 66	0.48	0.05	
Serine (Ser)	GR	0.88	1, 33	0.36	0.68	
	UM	24.40	2, 66	0.01 **	0.67	I > II, III; II < III
	GR × UM	23.15	2, 66	0.01 **	0.65	SI > SII, SIII; SII < SIII; SII < CII
Taurine (Tau)	GR	2.13	1, 33	0.16	0.15	
	UM	7.80	2, 66	0.01 **	0.39	I > III
	GR × UM	16.18	2, 66	0.01 **	0.57	SII < SI; CII > SII
Threonine (Thr)	GR	0.68	1, 33	0.51	0.03	
	UM	14.41	2, 66	0.01 **	0.55	II < I, III
	GR × UM	6.72	2, 66	0.01 **	0.30	SII < SI, SIII
Tryptophan (Trp)	GR	0.53	1, 33	0.45	0.04	
	UM	13.45	2, 66	0.01 **	0.52	II < I, III
	GR × UM	5.04	2, 66	0.01 *	0.29	SII < SI, SIII; CII > SII
Valine (Val)	GR	0.18	1, 33	0.67	0.01	II < I, III
	UM	13.9	2, 66	0.01 **	0.53	
	GR × UM	0.32	2, 66	0.32	0.03	

Note: Study design: GR, group; UM, ultramarathon; S, runners who received a single high dose of vitamin D; C, runners who received the placebo (control group); I, 24 h before the run; II, immediately after the run; and III, 24 h after the run. Significant difference detected at * *p* ≤ 0.05 or ** *p* ≤ 0.01.

**Table 2:** Two-way ANOVA (2 groups × 3 repeated measures) of changes in amino acid metabolite levels induced by the ultramarathon.

Variable	Effect	F	Df	*p*	Effect Size (η^2^)	Post Hoc Outcome
Asymmetric dimethylarginine (ADMA)	GR	0.32	1, 33	0.57	0.02	
	UM	12.10	2, 66	0.01 **	0.50	II < I, III; I < III
	GR × UM	3.12	2, 66	0.06	0.20	
Arginine to asymmetric dimethylarginine ratio (Arg/ADMA)	GR	4.11	1, 33	0.06	0.25	
	UM	28.11	2, 66	0.01 **	0.70	II < I, III; I > III
	GR × UM	23.96	2, 66	0.01 **	0.66	SII < SI, SIII; SI > SIII; CII > SII
Beta-Alanine (bAla)	GR	0.44	1, 33	0.51	0.03	
	UM	2.64	2, 66	0.09	0.18	
	GR × UM	2.65	2, 66	0.09	0.18	
Citrulline (Cit)	GR	0.35	1, 33	0.55	0.01	
	UM	6.01	2, 66	0.01 **	0.16	II < I
	GR × UM	8.02	2, 66	0.01 **	0.20	SII < SI, SIII; SI > SIII
Dimethylamine (DMA)	GR	8.18	1, 33	0.01 *	0.42	S > C
	UM	23.49	2, 66	0.01 **	0.68	II > I, III
	GR × UM	3.63	2, 66	0.04 *	0.24	SII > SI, SIII; SII > CII
Gamma-aminobutyric acid (GABA)	GR	0.08	1, 33	0.77	0.01	
	UM	0.54	2, 66	0.58	0.04	
	GR × UM	2.50	2, 66	0.10	0.17	
Homoarginine (hArg)	GR	0.34	1, 33	0.56	0.03	
	UM	7.43	2, 66	0.01 **	0.42	II < I, III; I > III
	GR × UM	0.29	2, 66	0.74	0.02	
Ornithine (Orn)	GR	2.58	1, 33	0.13	0.19	
	UM	27.29	2, 66	0.01 **	0.71	II < I, III; I > III
	GR × UM	5.41	2, 66	0.01 *	0.33	SII < SI, SIII; CII > SII
Sarcosine	GR	0.82	1, 33	0.38	0.06	
	UM	0.47	2, 66	0.62	0.03	
	GR × UM	3.13	2, 66	0.06	0.20	
Symmetric dimethylarginine (SDMA)	GR	2.59	1, 33	0.11	0.07	
	UM	44.56	2, 66	0.01 **	0.57	I < II, III
	GR × UM	3.96	2, 66	0.02 *	0.11	SI < SII, SIII; SIII < CIII
Tryptophan to branched-chain amino acid ratio (Trp/BCAA)	GR	0.79	1, 33	0.39	0.06	
	UM	2.52	2, 66	0.10	0.17	
	GR × UM	4.61	2, 66	0.02 *	0.27	CII > CI

Note: Note: Study design: GR, group; UM, ultramarathon; S, runners who received a single high dose of vitamin D; C, runners who received the placebo (control group); I, 24 h before the run; II, immediately after the run; and III, 24 h after the run. Significant difference detected at * *p* ≤ 0.05 or ** *p* ≤ 0.01.

## Data Availability

The data that support the findings of this study are available on request from the corresponding authors, J.M. and J.A.

## Supplementary Materials

The following supporting information can be downloaded at: https://www.mdpi.com/article/10.3390/nu15163536/s1, Table S1: Participants’ characteristics (*n* = 35); Table S2: Summary of training loads during typical one week of the two periods of training (*n* = 35); Table S3: Characteristics of the maximum oxygen uptake capacity (VO_2_ max) in ultramarathon runners based on the Cooper test (Cooper, 1968).
