# Supplementary material for: Vitamin D Supplementation Influences Ultramarathon-Induced Changes in Serum Amino Acid Levels, Tryptophan/Branched-Chain Amino Acid Ratio, and Arginine/Asymmetric Dimethylarginine Ratio
Source: Nutrients. 2023 Aug 11;15(16):3536. doi: 10.3390/nu15163536 (PMC10459061; doi:10.3390/nu15163536)
Supplement: Supplementary file 1 [file nutrients-15-03536-s001.zip › nutrients-2531091-supplementary.pdf]

Table S1. Participants' characteristics ($n = 35$)

Variable	Supplemented Group ($N = 16$) Mean ± SD	Control Group ($N = 19$) Mean ± SD	P	Effect Size (η^2)
Age (years)	42.40 ± 7.59	39.48 ± 6.89	0.21	0.04
Body height (cm)	175.20 ± 4.34 *	179.67 ± 4.64	0.01	0.17
Body mass	72.51 ± 6.71	76.19 ± 5.25	0.07	0.08
Body mass index (kg/m2)	23.24 ± 2.78	24.45 ± 1.19	0.11	0.06
Fat mass (%)	12.13 ± 3.89	12.85 ± 4.42	0.36	0.03

Note: *, significant difference vs. control group at $p < 0.05$.

Table S2. Summary of training loads during typical one week of the two periods of training ($n = 35$).

		Number Of Training Units Per Week	CR 1 (Km)	CR 2 (Km)	CROSS 1 (Km)	CROSS 2 (Km)	Speed (Km)
General preparation period	Mean	5.00	60.94	11.58	7.58	3.91	0.88
	SD	0.83	16.29	4.21	2.78	2.88	0.60
Pre-start preparation period	Mean	5.70	67.39	13.52	14.38	5.7	1.57
	SD	0.85	11.96	2.60	4.35	2.65	0.59

Note: CR 1–70–80% HR max - continuous running in the first intensity range (70–80% HRmax); CR 2–80–90% HR max - continuous running in the second intensity range (80–90% HRmax); CROSS 1 – up-downhill running in different tempo (75–85% HR max); CROSS 2–up-downhill running in different tempo (85-95% HR max); Speed–100–200 meters distance running with high intensity.

Table S3. Characteristics of the maximum oxygen uptake capacity (VO_2 max) in ultramarathon runners based on the Cooper test (Cooper, 1968).

Variable	Supplemented Group ($N = 16$) Mean ± SD	Control Group ($N = 19$) Mean ± SD	P	Effect Size (η^2)
VO_2max (ml × kg^{-1} × min^{-1})	53.73 ± 6.04	54.40 ± 5.68	0.74	<0.01
Distance (km)	2.908 ± 0.263	2.939 ± 0.254	0.75	<0.01

Note: VO_{2max} - maximal oxygen uptake
